# Group-based body psychotherapy improves appreciation of body awareness in post-treatment cancer patients: A non-randomized clinical trial

**DOI:** 10.3389/fpsyg.2023.956493

**Published:** 2023-04-06

**Authors:** Astrid Grossert, Cornelia Meffert, Viviane Hess, Christoph Rochlitz, Miklos Pless, Sabina Hunziker, Brigitta Wössmer, Ulfried Geuter, Gunther Meinlschmidt, Rainer Schaefert

**Affiliations:** ^1^Department of Psychosomatic Medicine, University Hospital Basel, Basel, Switzerland; ^2^Department of Medical Oncology, University Hospital Basel, Basel, Switzerland; ^3^Division of Clinical Psychology and Psychotherapy, Department of Psychology, University of Basel, Basel, Switzerland; ^4^Medical Center of Oncology and Hematology, Department of Psycho-Oncology, Cantonal Hospital Baselland, Liestal, Switzerland; ^5^Faculty of Medicine, University of Basel, Basel, Switzerland; ^6^Department of Medical Oncology, Winterthur Cantonal Hospital, Winterthur, Switzerland; ^7^Outpatient Clinic for Psychotherapy, Olten, Switzerland; ^8^Institute for Sports and Motology, University of Marburg, Marburg, Germany; ^9^Department of Digital and Blended Psychosomatics and Psychotherapy, Psychosomatic Medicine, University Hospital Basel, Basel, Switzerland; ^10^Division of Clinical Psychology and Cognitive Behavioral Therapy, International Psychoanalytic University Berlin, Berlin, Germany; ^11^Division of Clinical Psychology and Epidemiology, Department of Psychology, University of Basel, Basel, Switzerland

**Keywords:** body awareness, bodily disturbances, integrative body psychotherapy, malignant neoplasms, psychotherapy, Psycho-Oncology

## Abstract

**Introduction:**

Cancer-related impairments often co-occur with bodily disturbances. Body psychotherapy (BPT) can improve bodily wellbeing, yet evidence in cancer survivors is scarce. Hence, we aimed to evaluate whether blended group BPT alleviates bodily disturbances in post-treatment cancer patients.

**Methods:**

We conducted a bi-center study (registered in ClinicalTrials.gov, under No. NCT03707548), applying a pre-post convergent parallel design of weekly group BPT interspersed with smartphone-based ambulatory interventions using a waiting-period comparator. We included patients with completed curatively intended treatment for malignant neoplasms, suffering from bodily disturbances. The primary outcome was body image disturbances. Secondary outcomes were experiencing and appreciating body awareness, mental wellbeing, and health-related quality of life.

**Results:**

Forty patients (mean age 51.7 years) attended group BPT. Mixed-effect linear regression models contrasting intervention with the waiting period did not show statistically significant differences regarding the primary outcome [Pre-post difference contrasts: 1.44, 95% confidence interval (CI): −1.51 to 4.93, *p* = 0.339]. However, patients showed greater improvements in appreciating body awareness, measured by the “Body Mindfulness Questionnaire” (BMQ), from pre- to post-intervention as compared to the waiting period (pre-post difference contrasts: 7.31 95% CI: 4.15–10.47, Bonferroni-Holm corrected *q* = 0.0002).

**Discussion:**

We found no evidence that blended group BPT was effective in improving body image disturbances in post-treatment cancer patients, but found indications for an increase in body awareness appreciation.

**Clinical trial registration:**

ClinicalTrials.gov, identifier NCT03707548.

## 1. Introduction

The diagnosis of cancer often leads to high levels of distress in patients, with half of all cancer patients experiencing clinically relevant psychosocial distress (Mehnert et al., 2014). Cancer treatments can also cause physical and emotional changes, which can affect patients’ appearance, bodily functions, and autonomy, leading to body image concerns; These changes can include hair loss, scars, skin irritations, amputations, limited energy and performance, and sexual issues (Lerro et al., 2012; Fitch et al., 2021). Body image disturbances due to cancer and its treatments are a significant biopsychosocial impairment (Taylor-Ford et al., 2013; Rhoten et al., 2014; Rhondali et al., 2015; Benedict et al., 2016; Boquiren et al., 2016; Esser et al., 2018). Notably, they affect a substantial number of patients (Fingeret et al., 2014), while many of them express dissatisfaction with care received due to body image disturbance issues (Fingeret et al., 2012). We use the definition of Rhoten and colleagues (Rhoten, 2016) to define “body image disturbances,” which encompass perceptive, affective, and cognitive components and are a relevant and appropriate reflection of significant changes related to cancer. While developing the definition of body image disturbances, the authors identified key aspects that included self-perception of changes in appearance, displeasure with those changes, decline in physical functioning, and psychological distress caused by these changes. Despite the general challenge of a verbalized definition of body experience, commonly observed phenomena that accompany deterioration in experiencing one’s body are anxiety, social withdrawal, depressive tendencies, shame, inadequacy, and altered access to intimacy and sexuality (Lehmann et al., 2015; Kołodziejczyk and Pawłowski, 2019; Esplen and Fingeret, 2021; Bowie et al., 2022). It’s important to note that when referring to body image, it can mean both an objective observation of the body and a subjective, complex-multidimensional verbal-non-verbal reference to one’s own body (Joraschky and Pöhlmann, 2017).

Studies exploring the impact of cancer and its treatment on patients’ body experiences show a heterogeneous prevalence of body image disturbances in cancer patients, ranging from about one-third up to 80%, depending on the cancer type and applied assessment instrument, such as the Body Image Scale (BIS) (Fobair et al., 2006; Dahl et al., 2010; Fingeret et al., 2012; Rhondali et al., 2015; Benedict et al., 2016).

There are various interventions available to assist individuals struggling with body image issues. A review by Alleva et al. (2015) revealed that cognitive behavioral therapy (CBT)-based interventions on their own had a small to moderate effect size across indications. Additionally, exercise has been linked to improved body image across indications, as observed by Campbell and Hausenblas (2009). In patients with eating disorders, CBT-based interventions were effective in enhancing body image (Farrell et al., 2006). However, CBT in either a group or one-on-one setting for cancer patients has had mixed results (Fingeret et al., 2014), as has a wide range of interventions for female breast-cancer survivors (Morales-Sánchez et al., 2021). Esplen and colleagues found that the ReBIC (Restoring Body Image after Cancer) group program for breast cancer survivors was effective. This manual-based intervention included expressive exercises and guided imagery, integrated into a model that followed group therapy principles. It not only reduced patient distress regarding bodily disturbances but also led to long-lasting improvements in quality of life (Esplen et al., 2018).

There is increasing evidence supporting the effectiveness of body psychotherapy (BPT) in treating bodily disturbances, as noted by Koemeda-Lutz et al. (2006) and Rohricht et al. (2013). However, limited research has been conducted on the efficacy of BPT in cancer patients (Sollmann, 2000; Grossert et al., 2017). In response to this gap, we developed and implemented a group body psychotherapy program for post-treatment cancer patients, known as “KPTK: Körperpsychotherapie bei Krebs” (Grossert et al., 2017). Our approach was based on BPT, which is an experiential and holistic approach (Geuter, 2015; Kaul and Fischer, 2016). It intends to support patients who had completed therapy at least 3 months prior and were still experiencing body image disturbances despite successful treatment, by helping them coping with unwanted sensations and feelings related to their external appearance and body image (Fingeret et al., 2014) and altering their attitudes toward their body (Snobohm et al., 2010). This included addressing feelings of insecurity, vulnerability, stigmatization, impaired functioning, and disconnectedness from their body (Lindwall and Bergbom, 2009; Ervik and Asplund, 2012; Sekse et al., 2013; Boquiren et al., 2016; Esser et al., 2018). To facilitate the transfer of therapy into daily life, we provided patients with daily smartphone-based exercises between group therapy sessions.

The goal of our study was to assess the effectiveness of a blended intervention, combining group body psychotherapy (BPT) with smartphone-based ambulatory interventions, in mitigating body image disturbances resulting from cancer and its treatments. We hypothesized that post-treatment cancer patients would experience improved body image disturbances following the intervention compared to a waiting-period comparator. Additionally, we examined whether the intervention was linked to improvements in patients’ body awareness, mental wellbeing (including anxiety, depression, and suicidal tendencies), somatization, pain, and health-related quality of life.

## 2. Materials and methods

### 2.1. Study design, setting, and ethical issues

The study protocol of this trial, including methodological details, is published elsewhere (Grossert et al., 2019). We conducted this bi-center study, by applying a pre-post convergent parallel design of weekly group BPT interspersed with smartphone-based ambulatory interventions using a waiting-period comparator. All participants were scheduled to receive the same number and content of weekly group BPT, interspersed with the same number of daily smartphone-based interventions, in form of smartphone-based bodily interventions (15 in total) and control interventions (15 in total), while only the order in which the two types of smartphone-based interventions were provided varied each week, based on within-subject randomization with weekly blocks. The evaluation of short-term changes to these nested randomized smartphone-based interventions is not part of this paper, but about to be reported elsewhere. We recruited study participants from two hospitals in Switzerland (University Hospital Basel and Cantonal Hospital Winterthur). The *Ethikkommission Nordwest- und Zentralschweiz* granted ethical approval (EKNZ 2018-01115, date: 28 August 2018 and amendment dated 14 March 2019). Additionally, ethical approval was obtained from the *Kantonale Ethikkommission Zürich*. All participants signed an informed consent form before study participation. The study has been registered in ClinicalTrials.gov (NCT03707548), first registration on 16/10/2018. We assert that all procedures contributing to this work comply with the ethical standards of the relevant national and institutional committees on human experimentation and with the Helsinki Declaration of 1975, as revised in 2008.

### 2.2. Inclusion criteria and recruitment

Between September 3, 2018, and May 12, 2019, we included adult patients (age≥ 18 years, German speaking) who had received curatively intended treatment for any malignant neoplasm and were suffering from body image disturbances. Primary treatment had to be completed at least 3 months before recruitment. Bodily disturbances were defined as followed: Body image disturbances [Body Image Scale (BIS) ≥ 10] OR {(BIS = 2–9) AND patient-assessed distress due to bodily changes [Visual Analog Scale (VAS)-2 ≥ 5 out of 10]} OR {[therapist-assessed awareness of bodily changes (VAS-1 ≥ 5 out of 10) AND therapist-assessed related distress due to bodily changes (VAS-2 ≥ 5 out of 10)]}.

Furthermore, participants had to meet the following inclusion criteria: (1) no sign of progress or recurrence of malignancy at study inclusion, (2) an Eastern Cooperative Oncology Group (ECOG) performance score of 0–1, (3) an anticipated life expectancy of ≥12 months, and (4) the capacity to participate in 6 group-BPT sessions, two study assessments, and the smartphone-triggered interventions. Exclusion criteria were (1) suffering from a severe current mental disorder, (2) risk of current suicidality (as indicated by a suicide item score ≥2 in the Beck Depression Inventory) (Beck et al., 1996), (3) participation in any other clinical trial with a psychosocial intervention, and (4) receiving any other current psychotherapeutic treatment (except already existing therapies lasting ≥6 months). Recruitment took place at the two study centers (University Hospital Basel and the Cantonal Hospital Winterthur) and patients were additionally approached *via* public advertisements (e.g., advertisements in public transport and on the website of the Basel Cancer League).

### 2.3. Intervention

The intervention consisted of two elements: Patients participated in six group BPT sessions, 90 min each. In parallel, they received daily homework *via* smartphone. The manualized group BPT was carried out in small groups (range of 5–7 patients) and was provided by trained psychotherapists. For a detailed tabulated description of the intervention, we kindly refer to Grossert and colleagues (Grossert et al., 2019 #1361). The 6 group BPT sessions covered the following topics: The first session included a (1) general introduction, promoting group cohesion and focusing on body awareness; the second session (2) focused on physical resources and grounding, whereas the third session (3) had the focus on regulating closeness and distance. Session four (4) focused on social interactions and bodily impulses and session five (5) on embodied emotions. The sixth and last session of the group BPT intervention program (6) summarized the previous sessions and focused on the transferability of the content into daily life. The BPT group provided a protected frame for exploration and experimentation. Thereby, all sessions aimed to work in a supportive and resourceful way and to support the participants in developing strategies for facing cancer and its treatment. All group sessions followed the following three-step process: (1) The group sessions started with a body exercise to facilitate the arrival in the group and the own body. This was followed by a talking round to verbalize own experiences and sensations, including making reference to the past session where applicable. (2) The main part of the session addressed the translation of the session’s topic (see above) into body language and expression. After an experience-based input, the participants got the opportunity to describe their experiences in the group and to benefit from the commentaries of the other participants. (3) The third part of the sessions consisted of the closure of the session and an outlook on the upcoming week. The narrative and reflective parts at the beginning and end of each group session took place sitting in a circle, depending on the possibilities of the participants either on a chair or on a seat cushion.

The group BPT was provided as part of the outpatient service of University Hospital Basel and Cantonal Hospital Winterthur, using facilities from the Cancer Leagues Basel and Zuürich close to the hospitals. The therapist’s adherence to the manual was recorded with a respective checklist adapted to the session’s context (rated on a 4-point scale from 0 = “not at all” to 3 = “very accurate”). A senior body psychotherapist provided continuous supervision of the therapy.

As homework, participants received in a within-subject randomized fashion either a bodily intervention (3 times a week) or a control intervention (3 times a week) *via* smartphone over a period of five consecutive weeks. Hence, each participant was scheduled to receive the same number of smartphone-based bodily interventions (15 in total) and control interventions (15 in total). The smartphone-based bodily intervention offered audio clips consisting of BPT tools, experiences, and strategies. The control interventions consisted of 15 selected Grimm fairy tales improve intervention adherence, we contacted participants who did not turn up for a group appointment without having given prior notice. We asked patients to contact us at any time if they felt uncertain or had questions. Patients who attended fewer than 4 sessions were classified as dropouts.

### 2.4. Assessments

Patients were screened for eligibility at a baseline assessment (T0), using standardized questionnaires and a semi-structured interview. Included patients underwent a waiting period of approximately 6 weeks followed by a pre-intervention assessment (T1) and by weekly assessments after each group BPT session. After completion of the group BPT phase, the post-intervention assessment (T2) with standardized questionnaires and a semi-structured interview took place. Socio-demographic variables were assessed at T0, and medical variables at all time points, both were self-reported.

The primary endpoint, body image disturbances, was assessed using the “Body Image Scale” (BIS), which is a brief 10-item scale validated in cancer patients, showing high reliability (Cronbach’s alpha 0.93) and validity as well as sensitivity to change (Hopwood et al., 2001). On a four-point Likert scale, patients rated the extent to which they agreed with statements, such as “Have you been feeling self-conscious about your appearance?” (0 = “not at all” to 3 = “very much”). The summed total score ranges from 0 (“no distress”) to 30 (“high body image distress”). We translated this questionnaire from English into German according to the European Social Survey Translation Guidelines (Dorer, 2012).

Body awareness was measured at all three time points using the “Body Mindfulness Questionnaire” (BMQ) (Burg et al., 2017). The BMQ includes 14 items, such as “I forget my body in everyday stress.” It contains two subscales: “Experiencing Body Awareness” and “Appreciating Body Awareness.” Subscales are scored on a range from 7 to 42 with higher scores indicating a better outcome.

Furthermore, secondary outcomes were assessed using the “Somatic Symptom Disorder-B Criteria Scale” (SSD-12) (Toussaint et al., 2016), the “Hospital Anxiety and Depression Scale” (HADS) (Zigmond and Snaith, 1983), and the “Multidimensional Mood Questionnaire” (MDMQ) (Steyer, 2014). Quality of life was assessed using the “European Organization for Research and Treatment of Cancer” (EORTC QLQ-C30) questionnaire (Aaronson et al., 1993) and two scales (Vitality and Mental Health) of the “Short Form Health Survey” (SF-36) (Ware, 2000). The “Beck Depression Inventory” (Beck et al., 1996) was used to assess suicidal tendencies. Additional information was collected using the “National Comprehensive Cancer Network (NCCN) Distress Thermometer” (DT) (Mehnert et al., 2006), and *via* two self-developed single item VAS (0–10) to assess “the deterioration of body image due to the disease” (VAS-1) and “the degree of suffering from that deterioration” (VAS-2), with the two VAS independently filled in by patient and therapist. Furthermore, the assessment included two semi-standardized individual face-to-face interviews (30–50 min) at baseline and after completion of the group BPT phase, which were audiotaped if participants provided informed consent. In the baseline interview, patients were screened for whether they were eligible for the BPT intervention or not. The final interview clarified the acceptance and burden of the intervention as well as the need for further psychological support.

### 2.5. Statistical analyses

The sample size of the planned project was based on an *a priori* power analysis, using the software G*Power (Faul et al., 2007). In the absence of more specific previous published evidence to inform more specific effect-size assumptions, we chose a medium effect size that we deemed clinically relevant: With 52 participants completing the group BPT, we estimated to have sufficient power (1-β = 0.94) to describe pre-post differences of medium effect size (*d* = 0.5) in the primary outcome. With a 30% dropout rate and a safety margin of 10% accounting for unexpected variation in our estimates, we aimed at including a total of *N* = 88 patients.

We compared dropouts and patients who remained in the study until follow-up, looking at the variables age, gender, and distress at baseline (DT, VAS-1, and VAS-2) using *t*-tests for independent samples and χ2 tests in case of nominal data.

We checked the data for normal distributions by histograms and qq-plots. We used descriptive analyses for sample characteristics. Efficacy analyses were conducted *via* mixed modeling using restricted maximum likelihood (REML) estimation, with each outcome measure being entered as an outcome variable in a separate mixed-effect linear regression model, contrasting changes from T0 to T1 with changes from T1 to T2. The effect of time (days between T0 and T1 and days between T1 and T2) was entered in each model. As additional random effects, we entered study site, group, and case. Our primary analysis was adjusted for the covariates gender and age, but we also computed crude analyses. As indicators of model fit, we calculated Akaike’s Information Criterion. For the main mixed model analyses, we included all patients enrolled in the study [intention-to-treat (ITT) analyses]. Per protocol (PP) analyses were also conducted using data from participants who completed all group BPT sessions. The mixed models addressed missing values. We performed Bonferroni-Holm corrections to reduce the alpha error resulting from multiple statistical comparisons of secondary outcomes. All analyses were carried out using IBM SPSS Statistics, version 22 (SPSS Inc., Chicago, IL, USA) or STATA 15 (Stata Corp., College Station, TX, USA) statistical software.

## 3. Results

### 3.1. Sample descriptives and feasibility analyses

**FIGURE 1:**
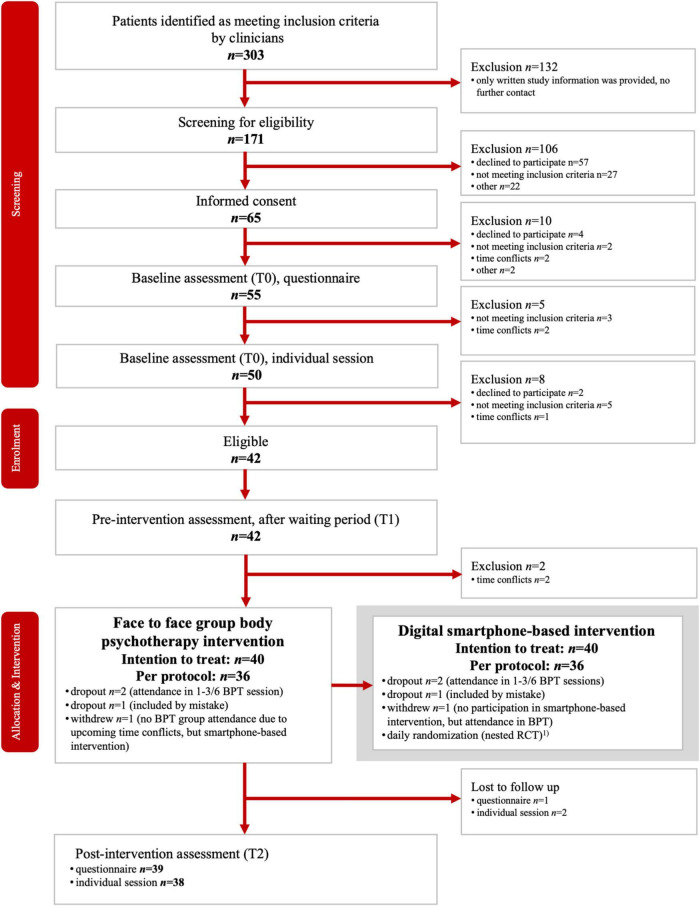
Study flow-chart. The smartphone-triggered bodily and control interventions were provided over a period of 5 consecutive weeks on 6 days per week, in parallel to the BPT sessions. Thus, each patient underwent 15 bodily and 15 control interventions. The results are not part of this paper and will be reported elsewhere.

We screened 171 patients, of whom 40 were allocated to the intervention (see Flow Chart, Figure 1). Thirty-nine patients met the inclusion criteria; one patient was included incorrectly and is part of the ITT analysis, exclusively. Patient characteristics are presented in Table 1.

**TABLE 1:** Sample characteristics.

Characteristics	Intention to treat (*N* = 40)		Per protocol (*n* = 36)	
	*N*	%	*N*	%
**Sex:**				
Female	35	87.5	32	88.9
Male	5	12.5	4	11.1
**Level of education^a^:**				
Elementary school	8	20.5	8	22.9
Secondary school	12	30.8	11	31.4
Technical college entrance qualification	8	20.5	5	14.3
High school graduation	8	20.5	8	22.9
Other certificate	3	7.7	3	8.6
**Main diagnosis:**				
MN of breast	23	57.5	22	61.1
Hodgkin lymphoma	4	10.0	4	10.9
Non-Hodgkin lymphoma	3	7.5	2	5.6
MN of lung	2	5.0	2	5.6
MN of ovary	1	2.5	1	2.8
MN of testis	1	2.5	1	2.8
MN of rectum	1	2.5	1	2.8
MN of small intestine	1	2.5	–	–
MN of tongue	1	2.5	1	2.8
MN of kidney cell	1	2.5	–	–
MN of stomach	1	2.5	1	2.8
MN of peritoneum	1	2.5	1	2.8
	Mean	SD	Mean	SD
Age (range 22–77 years)	51.7 years	13.8	51.8 years	14.4
Deterioration of body image due to the disease (VAS-1) ^b^	5.43	2.40	5.33	2.32
How much do you suffer from it? (VAS-2)^b^	5.38	2.33	5.28	2.24
NCCN Distress Thermometer^b^	5.80	1.90	5.72	1.86

SD, standard deviation; MN, malignant neoplasm; NCCN, national comprehensive cancer network; VAS, Visual Analog Scale.

^a^Totals that do not add up to N = 40 / n = 36 are due to missing values.

^b^Baseline values range from 0 to 10, with 10 representing higher burden.

Seven BPT groups (4 in Basel, 3 in Winterthur) consisting of 5–7 patients were carried out. Each group received 6 sessions within a period of 6–9 weeks (due to public holidays and room allocation). The waiting period between T0 and T1 was significantly shorter, with a median of 33.0 days [interquartile range (IQR) 22.0–58.5 days] than the time of the group BPT between T1 and T2, with a median of 58.0 days (IQR 49.0–62.0 days; *p* = 0.030).

The therapists’ overall adherence to the intervention manual was very good (*M* = 2.70; range between sessions *M* = 2.59 to *M* = 2.91). Patients’ adherence to the intervention was high, as 92.5% (*n* = 37) of the included participants attended at least 4 BPT sessions. Two patients were classified as dropouts. One patient did not participate in the sessions due to time conflicts but participated in the smartphone-based intervention. These three patients and the one patient who was included incorrectly were excluded from the PP analysis.

The non-participant and the two patients who dropped out during the study showed higher distress on the DT than patients who completed the group BPT (*M* = 7.7, *SD* = 0.6 vs. *M* = 5.7, *SD* = 1.9; *p* = 0.003). Apart from this, patients in these two groups neither differed significantly in terms of age (*p* = 0.866) nor gender (*p* = 0.338).

### 3.2. Efficacy analyses

The summary statistics of all primary and secondary outcomes at all three time points are shown in Table 2.

**TABLE 2:** Summary statistics of all primary and secondary outcomes at all three time points.

Assessment instruments (range)	T0			T1			T2		
	*N*	Mean	SD	*N*	Mean	SD	*N*	Mean	SD
Body Image Scale (BIS) (0–30, higher values indicate higher burden)	40	11.01	6.30	40	9.66	5.38	38	9.74	5.78
BMQ experiencing body awareness (7–42, higher values represent a better outcome)	39	23.34	6.07	39	22.59	6.58	38	23.45	7.90
BMQ appreciating body awareness (7–42, higher values represent a better outcome)	39	29.90	7.28	39	28.58	7.39	38	33.37	8.91
HADS anxiety (0–21, higher values indicate higher severity)	40	9.28	3.06	40	8.78	3.39	38	8.53	3.42
HADS depression (0–21, higher values indicate higher severity)	40	7.18	3.49	40	6.83	4.09	38	6.40	4.52
SF-36 vitality (0–100, higher values represent a better outcome)	40	41.67	17.21	40	41.67	17.33	39	45.51	20.61
SF-36 mental health (0–100, higher values represent a better outcome)	40	57.40	13.05	40	59.90	15.81	39	61.10	18.26
EORTC QLQ-C30 global health status (0–100, higher values represent a higher level of functioning)	40	57.92	15.21	40	58.54	15.39	38	63.82	18.20
EORTC QLQ-C30 physical functioning (0–100, higher values represent a higher level of functioning)	40	78.17	15.17	40	77.67	16.09	38	82.54	14.15
EORTC QLQ-C30 role functioning (0–100, higher values represent a higher level of functioning)	40	52.08	28.54	40	55.00	27.79	38	59.65	28.38
EORTC QLQ-C30 emotional functioning (0–100, higher values represent a higher level of functioning)	40	42.50	22.07	40	45.42	22.95	39	47.65	23.41
EORTC QLQ-C30 cognitive functioning (0–100, higher values represent a higher level of functioning)	40	54.17	23.19	40	55.83	27.10	39	58.97	24.44
EORTC QLQ-C30 social functioning (0–100, higher values represent a higher level of functioning)	40	55.00	30.01	40	51.67	30.62	39	55.56	30.91
EORTC QLQ-C30 fatigue (0–100, higher values indicate higher burden)	40	56.67	25.01	40	54.44	24.50	39	53.28	23.53
EORTC QLQ-C30 nausea and vomiting (0–100, higher values indicate higher burden)	40	6.25	9.76	40	6.25	12.34	39	5.13	17.59
EORTC QLQ-C30 pain (0–100, higher values indicate higher burden)	40	37.92	25.60	40	41.67	25.60	39	44.02	28.22
EORTC QLQ-C30 dyspnea (0–100, higher values indicate higher burden)	39	35.04	34.16	40	30.83	30.56	38	28.07	26.31
EORTC QLQ-C30 insomnia (0–100, higher values indicate higher burden)	40	57.50	28.23	40	57.50	33.75	39	52.14	31.34
EORTC QLQ-C30 appetite loss (0–100, higher values indicate higher burden)	40	15.83	25.02	40	11.67	22.07	39	13.68	21.25
EORTC QLQ-C30 constipation (0–100, higher values indicate higher burden)	40	10.00	17.21	40	10.83	19.08	39	10.26	20.45
EORTC QLQ-C30 diarrhea (0–100, higher values indicate higher burden)	40	18.33	30.15	40	17.50	28.23	39	19.66	28.32
SSD-12 total score (0–42, higher values indicate higher severity; subscales 0–16)	39	24.15	8.20	40	23.28	6.69	38	21.27	7.53
SSD-12 subscale cognitive aspects	39	6.75	2.66	40	6.55	2.22	38	6.43	2.24
SSD-12 subscale affective aspects	40	9.45	3.51	40	9.35	2.70	38	8.37	2.81
SSD-12 subscale behavioral aspects	40	7.51	3.39	40	7.38	2.75	38	6.47	3.52
NCCN distress thermometer (0–10, higher values indicate higher severity)	40	5.80	1.90	40	5.58	2.21	39	4.51	2.33

T0 = baseline, T1 = after the waiting period, T2 = end of participation; SD, standard deviation; BIS, Body Image Scale; BMQ, body mindfulness questionnaire; HADS, Hospital Anxiety and Depression Scale; SF-36, 36 item short form health survey; EORTC, European Organization for Research and Treatment of Cancer; QLQ, quality of life questionnaire; SSD-12, Somatic Symptom Disorder-B Criteria Scale; NCCN, national comprehensive cancer network.

Results from the adjusted mixed-effect linear regression models contrasting the intervention period (T1–T2) with the waiting period (T0–T1) are presented in Table 3. Regarding changes from pre- to post-intervention, treatment effects concerning the primary endpoint body image were statistically not significant (BIS: *p* = 0.339; 95% CI: −1.51, 4.93). Patients showed greater improvements in appreciating body awareness during the intervention period as compared to the waiting period (BMQ: Bonferroni-Holm corrected *q* < 0.001; 95% CI: 4.15, 10.47). In other secondary endpoints, there were no statistically significant differences between the intervention period and the waiting period. Results of respective unadjusted, crude analyses are provided as Supplementary Table 1. The per protocol or completer analyses led to comparable results, which we provide as Supplementary material (see Supplementary Tables 2, 3). Suicidal tendencies, which were one of the secondary outcomes, could not be modeled statistically due to a lack of dispersion of values. Only one patient stated at time point T1 that “he would have committed suicide if he had been able to do so,” but showed no current or further suicidal tendencies.

**TABLE 3:** Results from mixed-effect linear regression models contrasting the intervention period (T1 to T2) with the waiting period (T0 to T1), adjusted by gender and age.

Adjusted analyses (intention-to-treat, adjusted by gender and age)	Contrast (SE)		Esti-mate	SE	95% CI		*p*-value	*q*-value
	T1 vs. T0	T2 vs. T1			LB	UB		
Body Image Scale (BIS) (0–30, higher values indicate higher burden)	−1.42 (1.18)	0.02 (0.83)	1.44	1.51	−1.51	4.39	0.339026497	1
BMQ experiencing body awareness (7–42, higher values represent a better outcome)	−2.55 (1.32)	0.35 (0.93)	2.90	1.69	−0.41	6.21	0.086191176	1
BMQ appreciating body awareness (7–42, higher values represent a better outcome)	−3.02 (1.27)	4.29 (0.89)	7.31	1.61	4.15	10.47	5.95192E-06	0.0002
HADS anxiety (0–21, higher values indicate higher severity)	−0.94 (0.65)	−0.45 (0.46)	0.49	0.82	−1.12	2.10	0.553570061	1
HADS depression (0–21, higher values indicate higher severity)	−0.07 (0.53)	−0.36 (0.38)	−0.29	0.68	−1.61	1.04	0.6731611	1
SF-36 vitality (0–100, higher values represent a better outcome)	2.75 (3.52)	4.53 (2.46)	1.78	4.47	−6.99	10.54	0.690924705	1
SF-36 mental health (0–100, higher values represent a better outcome)	0.58 (3.53)	0.45 (2.49)	−0.14	4.51	−8.98	8.71	0.97606971	1
EORTC QLQ-C30 global health status (0–100, higher values represent a better outcome)	−2.01 (3.80)	4.87 (2.70)	6.88	4.88	−2.68	16.45	0.15858322	1
EORTC QLQ-C30 physical functioning (0–100, higher values represent a better outcome)	0.16 (2.29)	4.92 (1.60)	4.76	2.90	−0.93	10.45	0.101088126	1
EORTC QLQ-C30 role functioning (0–100, higher values represent a better outcome)	2.92 (5.81)	4.13 (4.10)	1.21	7.42	−13.32	15.75	0.870113172	1
EORTC QLQ-C30 emotional functioning (0–100, higher values represent a better outcome)	−5.10 (5.08)	0.05 (3.57)	5.15	6.47	−7.53	17.83	0.425726594	1
EORTC QLQ-C30 cognitive functioning (0–100, higher values represent a better outcome)	−3.45 (5.43)	1.93 (3.81)	5.39	6.92	−8.17	18.94	0.435943921	1
EORTC QLQ-C30 social functioning (0–100, higher values represent a better outcome)	−5.57 (5.62)	2.47 (3.95)	8.04	7.16	−5.99	22.06	0.261263259	1
EORTC QLQ-C30 fatigue (0–100, higher values indicate higher burden)	−5.33 (5.08)	−1.79 (3.57)	3.54	6.47	−9.14	16.23	0.584136013	1
EORTC QLQ-C30 nausea and vomiting (0–100, higher values indicate higher burden)	−0.39 (4.02)	−1.15 (2.92)	−0.76	5.26	−11.07	9.55	0.88529742	1
EORTC QLQ-C30 pain (0–100, higher values indicate higher burden)	2.46 (6.10)	1.78 (4.30)	−0.68	7.80	−15.95	14.60	0.930960732	1
EORTC QLQ-C30 dyspnea (0–100, higher values indicate higher burden)	−0.84 (6.04)	−1.17 (4.25)	−0.33	7.66	−15.34	14.69	0.966067436	1
EORTC QLQ-C30 insomnia (0–100, higher values indicate higher burden)	4.80 (7.41)	−3.75 (5.23)	−8.55	9.47	−27.11	10.01	0.36671057	1
EORTC QLQ-C30 appetite loss (0–100, higher values indicate higher burden)	2.52 (5.69)	4.17 (4.03)	1.65	7.29	−12.64	15.94	0.821284523	1
EORTC QLQ-C30 constipation (0–100, higher values indicate higher burden)	1.12 (4.93)	−0.42 (3.51)	−1.54	6.34	−13.97	10.90	0.808740213	1
EORTC QLQ-C30 diarrhea (0–100, higher values indicate higher burden)	6.99 (5.11)	4.44 (3.57)	−2.55	6.48	−15.26	10.16	0.694018886	1
SSD-12 total score (0–42, higher values indicate higher severity; subscales 0–16)	−0.49 (1.43)	−1.87 (1.00)	−1.38	1.82	−4.94	2.18	0.447071517	1
SSD-12 subscale cognitive aspects	0.15 (0.55)	−0.01 (0.39)	−0.15	0.71	−1.54	1.23	0.829019175	1
SSD-12 Subscale Affective aspects	−0.32 (0.70)	−1.03 (0.49)	−0.71	0.89	−2.46	1.04	0.424934518	1
SSD-12 subscale behavioral aspects	0.16 (0.55)	−0.82 (0.39)	−0.99	0.70	−2.36	0.39	0.161386963	1
NCCN distress thermometer (0–10, higher values indicate higher severity)	−0.37 (0.52)	−1.06 (0.37)	−0.69	0.66	−1.99	0.61	0.298341525	1

T0 = baseline, T1 = after the waiting period, T2 = end of participation; SE, standard error; CI, confidence interval; LB, lower-bound; UB, upper-bound; q-value: adjusted p-value (Bonferroni-Holm); BIS, body image scale; BMQ, body mindfulness questionnaire; HADS, Hospital Anxiety and Depression Scale; SF-36, 36 item short form health survey; EORTC, European Organization for Research and Treatment of Cancer; QLQ, quality of life questionnaire; SSD-12, Somatic Symptom Disorder-B Criteria Scale; NCCN, national comprehensive cancer network.

## 4. Discussion

Our study aimed at evaluating the effects of group BPT on subjects with cancer-related body image disturbances. Neither the ITT nor the PP analyses indicated any significant effects of group BPT regarding our primary outcome “body image disturbance” and most secondary outcomes. However, strong intervention effects and significant improvements were observed with regard to the appreciation of body awareness, as a secondary outcome.

Several factors may have contributed to our main result of no evidence for an effect of BPT on body image disturbance, including (i) insufficient statistical power of our study to capture small to medium effects, as it was not possible to recruit the intended number of participants, given a rather short recruitment period due to the limited funding duration, (ii) our six-session-BPT being too short to exert effects, and (iii) lack of a longer follow-up assessment period. Furthermore, body image disturbances are a multidimensional construct that includes objective and subjective elements. These are perceptions, feelings, and attitudes toward the body, such as loss of attractiveness and self-confidence, negative body judgment, accentuation of external appearance, worry about possible physical deficits, sexual problems, and the overall feeling of loss of wholeness (Carver et al., 1998). Similarly, body image disturbances due to cancer are multifaceted beyond what is captured with the BIS. Hence, more longitudinal studies are needed to investigate the effects of body-psychotherapeutic group interventions regarding the complex constructs of “body image disturbances” in specific, and “bodily disturbances” in general. Previous preliminary evidence from patients with heterogeneous somatoform disorders suggested that group body psychotherapy has the potential to reduce somatic symptoms (Rohricht et al., 2019). Notably, findings indicated that increasing self-acceptance related to body image, amongst others, may have acted as a mechanism for the observed change. This points out the potential of more holistic approaches, like body-oriented psychological interventions, to support patients’ abilities to cope, by enhancing self-acceptance of changes in bodily appearance and function. Indeed, one core aspect of our group intervention was to provide the patients the space to be mindful, without judgments, of the momentary experience of their own bodies.

We found statistically significant improvements from pre- to post-intervention in appreciating body awareness as compared to the waiting-period comparator, highlighting a specific effect of BPT on post-treatment cancer patients. We assessed “Appreciating Body Awareness” with a BMQ subscale. Yet, a value for a minimal clinically important difference has not been identified for that subscale, and further research is needed to address this question. Notably, the finding of improved appreciation of body awareness is in line with the concept of Integrative Body Psychotherapy (IBP) being an experience-oriented procedure that fosters access to the “felt body.” It thereby may trigger a senso-emotional-cognitive experience that can be expressed in the terms of Gendlin, as being able to form an intuitive body feel (“felt sense”) that can then provide the basis to move beyond the current situation or sense (“felt shift”) (Gendlin, 1982). Notably, an increase in appreciating body awareness may represent an intended treatment outcome in itself. Furthermore, it may trigger positive secondary effects. While it has been reported that increased body awareness is linked to reduced perceived stress, neuroticism, and depression (Rhondali et al., 2015), future studies should try identifying whether improvement in appreciation of body awareness goes along with long-term positive effects also in post-treatment cancer patients. In sum, to the best of our knowledge, this is the first investigation to show the efficacy of a brief BPT intervention regarding the appreciation of body awareness in cancer patients. Future studies with larger study populations and longer follow-up assessment periods are requested to confirm these results.

Our study has several limitations: First, we may not have captured all relevant aspects of body image disturbances, as we decided to use the BIS as an internationally renowned and well-established instrument that has been applied previously in studies with cancer patients. Notably, there are other larger and more comprehensive diagnostic tools available in German that may overcome this limitation, such as a 52-items questionnaire to assess peoples’ subjective views of their own bodies (*Fragebogen zur Beurteilung des eigenen Körpers*, FBeK) (Braehler et al., 2000) and the 35-items Dresden Body Image Inventory (DBIQ; *Dresden Körperbildfragebogen*, DKB-35) (Pöhlmann et al., 2014). Second, as yet, the BIS has no clearly defined cut-off value (see e.g., Melissant et al., 2018). Based on previous studies, a cut-off score of 10 or higher could be considered optimal for detecting the presence of clinically significant body image disturbances (Hopwood et al., 2000; Rhondali et al., 2015). Without knowing the actual clinical relevance of this cut-off, we used this value as an inclusion criterion. Third, it was not possible to recruit the intended number of participants, resulting in reduced statistical power. Therefore, our estimates had lower precision than originally anticipated. Fourth, our group BPT was designed for and open to all patients with any malignant neoplasm. Nevertheless, only five men participated and women with breast cancer represented most of the study population. This leaves uncertainty regarding the generalizability of the results, as we were unable to meaningfully repeat analyses after stratification for sex and cancer type to draw respective conclusions. In addition, the heterogeneity in patients and cancer type may have further diluted the effects estimated in this study, adding to limited statistical power noted above. Fifth, all participants were scheduled to receive the same number and content of daily smartphone-based interventions blended with the group BPT. Hence, our design did not allow us to contrast the potential effects of group BPT with and without the blended smartphone-based intervention component, which is a potential question for future studies. Sixth, without additional follow-up assessments, we were unable to determine the longer-term stability of changes.

Nevertheless, given that body disturbances affect subjects with different types of cancer, heterogeneity can also be considered a strength, as it allows for increasing the generalizability of findings to a broader range of cancer diagnoses. Of note, the intervention was well accepted, as indicated by a low dropout rate, pointing to the feasibility and acceptance of this type of treatment for post-treatment cancer survivors. Regarding the qualitative data collected *via* interview, we intend to analyze and publish them separately. We expect that the evaluation of these qualitative data will provide the opportunity to generate additional valuable insights, potentially informing future refinement and implementation of body psychotherapeutic interventions.

Despite successes in modern cancer therapy, many patients are suffering from cancer-related burdens and the consequences (Cleeland et al., 2013). Both, the disease itself and the therapy can leave physical and psychological marks. Symptom reduction is essential for patients with advanced disease. The results of our study suggest that while we found no evidence for group BPT regarding potential effects on body image disturbances, group BPT may be a suitable addition to the growing array of psychosocial interventions, which address the appreciation of body awareness of cancer patients. It could help patients returning to their normal lives. From a research perspective, our findings point out to the potential of elucidating appreciation of body awareness as an important aspect and outcome in the context of body psychotherapy.

Body image disturbances are highly relevant in cancer patients and may persist despite successful cancer therapies. They pose a major challenge to the wellbeing and quality of life of cancer patients and require to be addressed appropriately by care providers. This study did not find evidence for group BPT being effective in improving body image disturbances in post-treatment cancer patients. However, BPT may have the potential to foster the appreciation of body awareness following curative tumor therapy.

## Data availability statement

The datasets presented in this article are not readily available because of the nature of this research, participants of this study did not agree for their data to be shared publicly, so supporting data is not available. Requests to access the datasets should be directed to gunther.meinlschmidt@unibas.ch.

## Ethics statement

The studies involving human participants were reviewed and approved by the Ethikkommission Nordwest- und Zentralschweiz and the Kantonale Ethikkommission Zürich. The patients/participants provided their written informed consent to participate in this study.

## Author contributions

AG conceptualized the interventions and study design, obtained funding, and provided study materials, was one of two BPT therapists and took part in patients’ recruitment, study coordination, collection, and assembly of data and its interpretation, as well as the writing of the manuscript. CM participated in the entire coordination of the study, its design, collection, and assembly of data and its analysis and interpretation, and the writing of the manuscript. MP was responsible for the conduct of the study in Winterthur. BW, UG, VH, CR, and SH contributed to the study design and participated in obtaining funding. GM conceptualized the interventions and the study design, participated in obtaining funding, supervising the study, data assembly, analysis, interpretation, and writing of the manuscript. RS conceptualized the interventions and the study design, participated in obtaining funding, supervising the study and its coordination, data interpretation, and writing the manuscript. All authors read and approved the final manuscript.
