# Supplementary material for: Group-based body psychotherapy improves appreciation of body awareness in post-treatment cancer patients: A non-randomized clinical trial
Source: Front Psychol. 2023 Apr 6;14:956493. doi: 10.3389/fpsyg.2023.956493 (PMC10117640; doi:10.3389/fpsyg.2023.956493)
Supplement: Supplementary file 1 [file Data_Sheet_1.docx]

## **Supplementary Material**

**Supplementary Table 1.** Results from crude mixed-effect linear regression models contrasting T1 to T2 with T0 to T1

| **Crude analyses**  (Intention-to-treat) | Contrast (SE) | | Estimate | SE | 95% CI | | *p*-value | *q*-value |
| --- | --- | --- | --- | --- | --- | --- | --- | --- |
|  | T1 vs T0 | T2 vs T1 |  |  | LB | UB |  |  |
| Body Image Scale (BIS)  (0-30, higher values indicate higher burden) | -1.41 (1.18) | 0.03 (0.83) | 1.44 | 1.51 | -1.52 | 4.39 | 0.340012044 | 1 |
| BMQ Experiencing body awareness  (7-42, higher values represent a better outcome) | -2.61 (1.32) | 0.32 (0.93) | 2.93 | 1.69 | -0.38 | 6.24 | 0.082543562 | 1 |
| BMQ Appreciating body awareness  (7-42, higher values represent a better outcome) | -2.94 (1.27) | 4.33 (0.89) | 7.27 | 1.61 | 4.11 | 10.43 | 6.64657E-06 | 0.0002 |
| HADS Anxiety  (0-21, higher values indicate higher severity) | -0.95 (0.64) | -0.46 (0.46) | 0.49 | 0.82 | -1.12 | 2.10 | 0.549147081 | 1 |
| HADS Depression  (0-21, higher values indicate higher severity) | -0.10 (0.54) | -0.37 (0.38) | -0.28 | 0.68 | -1.61 | 1.05 | 0.680995453 | 1 |
| SF-36 Vitality  (0-100, higher values represent a better outcome) | 2.90 (3.52) | 4.59 (2.46) | 1.68 | 4.47 | -7.08 | 10.44 | 0.706596518 | 1 |
| SF-36 Mental Health  (0-100, higher values represent a better outcome) | 0.95 (3.53) | 0.66 (2.49) | -0.30 | 4.51 | -9.14 | 8.55 | 0.947381663 | 1 |
| EORTC QLQ-C30 Global health status  (0-100, higher values represent a better outcome) | -1.73 (3.80) | 5.01 (2.70) | 6.74 | 4.88 | -2.82 | 16.31 | 0.167202928 | 1 |
| EORTC QLQ-C30 Physical functioning  (0-100, higher values represent a better outcome) | 0.21 (2.28) | 4.94 (1.60) | 4.73 | 2.90 | -0.96 | 10.41 | 0.103227042 | 1 |
| EORTC QLQ-C30 Role functioning  (0-100, higher values represent a better outcome) | 3.22 (5.81) | 4.26 (4.10) | 1.03 | 7.42 | -13.51 | 15.57 | 0.889465314 | 1 |
| EORTC QLQ-C30 Emotional functioning  (0-100, higher values represent a better outcome) | -4.87 (5.07) | 0.18 (3.56) | 5.06 | 6.47 | -7.62 | 17.73 | 0.434146368 | 1 |
| EORTC QLQ-C30 Cognitive functioning  (0-100, higher values represent a better outcome) | -3.36 (5.41) | 1.96 (3.81) | 5.32 | 6.91 | -8.23 | 18.87 | 0.441473781 | 1 |
| EORTC QLQ-C30 Social functioning  (0-100, higher values represent a better outcome) | -4.95 (5.65) | 2.84 (3.95) | 7.79 | 7.17 | -6.27 | 21.84 | 0.277587219 | 1 |
| EORTC QLQ-C30 Fatigue  (0-100, higher values indicate higher burden) | -5.52 (5.08) | -1.88 (3.57) | 3.63 | 6.47 | -9.06 | 16.32 | 0.574755037 | 1 |
| EORTC QLQ-C30 Nausea and vomiting  (0-100, higher values indicate higher burden) | -0.25 (4.00) | -1.05 (2.90) | -0.80 | 5.24 | -11.08 | 9.48 | 0.878572741 | 1 |
| EORTC QLQ-C30 Pain  (0-100, higher values indicate higher burden) | 2.31 (6.09) | 1.69 (4.30) | -0.61 | 7.79 | -15.89 | 14.66 | 0.937093595 | 1 |
| EORTC QLQ-C30 Dyspnea  (0-100, higher values indicate higher burden) | -1.36 (6.05) | -1.41 (4.25) | -0.04 | 7.67 | -15.08 | 14.99 | 0.995382023 | 1 |
| EORTC QLQ-C30 Insomnia  (0-100, higher values indicate higher burden) | 4.07 (7.39) | -4.07 (5.22) | -8.14 | 9.47 | -26.70 | 10.42 | 0.389994036 | 1 |
| EORTC QLQ-C30 Appetite loss  (0-100, higher values indicate higher burden) | 2.54 (5.67) | 4.21 (4.02) | 1.66 | 7.28 | -12.61 | 15.93 | 0.819222484 | 1 |
| EORTC QLQ-C30 Constipation  (0-100, higher values indicate higher burden) | 1.14 (4.92) | -0.41 (3.50) | -1.55 | 6.34 | -13.98 | 10.88 | 0.806694224 | 1 |
| EORTC QLQ-C30 Diarrhea  (0-100, higher values indicate higher burden) | 6.52 (5.11) | 4.19 (3.57) | -2.33 | 6.48 | -15.03 | 10.37 | 0.719208165 | 1 |
| SSD-12 Total score  (0-42, higher values indicate higher severity; subscales 0-16) | -0.63 (1.43) | -1.94 (1.00) | -1.31 | 1.82 | -4.87 | 2.26 | 0.472291612 | 1 |
| SSD-12 Subscale Cognitive aspects | 0.06 (0.55) | -0.03 (0.39) | -0.09 | 0.71 | -1.48 | 1.29 | 0.894521988 | 1 |
| SSD-12 Subscale Affective aspects | -0.36 (0.70) | -1.05 (0.49) | -0.69 | 0.89 | -2.44 | 1.06 | 0.440036826 | 1 |
| SSD-12 Subscale Behavioral aspects | 0.12 (0.55) | -0.85 (0.39) | -0.97 | 0.70 | -2.35 | 0.41 | 0.168808208 | 1 |
| NCCN Distress Thermometer  (0-10, higher values indicate higher severity) | -0.43 (0.52) | -1.09 (0.37) | -0.66 | 0.67 | -1.96 | 0.65 | 0.322477271 | 1 |

*T0 = baseline, T1 = after the waiting period, T2 = end of participation; SE: Standard Error; CI: Confidence Interval; LB: lower-bound; UB: upper-bound; q-value: adjusted p-value (Bonferroni-Holm); BIS: Body Image Scale; BMQ: Body Mindfulness Questionnaire; HADS: Hospital Anxiety and Depression Scale; SF-36: 36 Item Short Form Health Survey; EORTC: European Organization for Research and Treatment of Cancer; QLQ: Quality of Life Questionnaire; SSD-12: Somatic Symptom Disorder-B Criteria Scale; NCCN: National Comprehensive Cancer Network.*

**Supplementary Table 2.** Results from mixed-effect linear regression models contrasting T1 to T2 with T0 to T1 in the completer sample

| **Crude analyses**  (Completer) | Contrast (SE) | | Estimate | SE | 95% CI | | *p*-value | *q*-value |
| --- | --- | --- | --- | --- | --- | --- | --- | --- |
|  | T1 vs T0 | T2 vs T1 |  |  | LB | UB |  |  |
| Body Image Scale (BIS)  (0-30, higher values indicate higher burden) | -1.27 (1.24) | -0.37 (0.87) | 0.90 | 1.60 | -2.22 | 4.03 | 0.571714961 | 1 |
| BMQ Experiencing body awareness  (7-42, higher values represent a better outcome) | -2.72 (1.43) | 0.49 (1.01) | 3.21 | 1.85 | -0.42 | 6.84 | 0.082787309 | 1 |
| BMQ Appreciating body awareness  (7-42, higher values represent a better outcome) | -2.87 (1.31) | 4.91 (0.92) | 7.79 | 1.69 | 4.47 | 11.10 | 4.06742E-06 | 0.0001 |
| HADS Anxiety  (0-21, higher values indicate higher severity) | -0.64 (0.65) | -0.65 (0.46) | -0.01 | 0.84 | -1.66 | 1.63 | 0.987899746 | 1 |
| HADS Depression  (0-21, higher values indicate higher severity) | 0.24 (0.51) | -0.77 (0.36) | -1.01 | 0.65 | -2.28 | 0.27 | 0.122225775 | 1 |
| SF-36 Vitality  (0-100, higher values represent a better outcome) | 2.38 (3.76) | 5.11 (2.64) | 2.73 | 4.84 | -6.76 | 12.22 | 0.572639513 | 1 |
| SF-36 Mental Health  (0-100, higher values represent a better outcome) | -0.47 (3.47) | 2.59 (2.44) | 3.06 | 4.47 | -5.70 | 11.82 | 0.493878924 | 1 |
| EORTC QLQ-C30 Global health status  (0-100, higher values represent a better outcome) | -2.82 (3.76) | 7.18 (2.67) | 10.00 | 4.86 | 0.46 | 19.53 | 0.039867507 | 0.9568 |
| EORTC QLQ-C30 Physical functioning  (0-100, higher values represent a better outcome) | -0.61 (2.36) | 5.77 (1.66) | 6.39 | 3.04 | 0.43 | 12.34 | 0.035498756 | 0.8875 |
| EORTC QLQ-C30 Role functioning  (0-100, higher values represent a better outcome) | 0.88 (5.87) | 6.37 (4.15) | 5.50 | 7.57 | -9.34 | 20.34 | 0.467886433 | 1 |
| EORTC QLQ-C30 Emotional functioning  (0-100, higher values represent a better outcome) | -6.52 (5.19) | 2.38 (3.65) | 8.90 | 6.68 | -4.20 | 22.00 | 0.183163987 | 1 |
| EORTC QLQ-C30 Cognitive functioning  (0-100, higher values represent a better outcome) | -6.11 (5.60) | 3.11 (3.94) | 9.23 | 7.22 | -4.92 | 23.37 | 0.201037589 | 1 |
| EORTC QLQ-C30 Social functioning  (0-100, higher values represent a better outcome) | -7.15 (5.79) | 5.25 (4.05) | 12.40 | 7.42 | -2.15 | 26.95 | 0.094864855 | 1 |
| EORTC QLQ-C30 Fatigue  (0-100, higher values indicate higher burden) | -3.92 (5.33) | -2.66 (3.76) | 1.26 | 6.87 | -12.21 | 14.73 | 0.854369971 | 1 |
| EORTC QLQ-C30 Nausea and vomiting  (0-100, higher values indicate higher burden) | 0.64 (4.14) | -1.34 (3.03) | -1.98 | 5.51 | -12.78 | 8.81 | 0.718745678 | 1 |
| EORTC QLQ-C30 Pain  (0-100, higher values indicate higher burden) | 2.56 (6.57) | 1.67 (4.66) | -0.89 | 8.51 | -17.57 | 15.79 | 0.916506584 | 1 |
| EORTC QLQ-C30 Dyspnea  (0-100, higher values indicate higher burden) | 0.43 (6.08) | -3.79 (4.29) | -4.22 | 7.80 | -19.51 | 11.06 | 0.587995972 | 1 |
| EORTC QLQ-C30 Insomnia  (0-100, higher values indicate higher burden) | 4.98 (7.91) | -4.47 (5.60) | -9.45 | 10.24 | -29.52 | 10.63 | 0.356322822 | 1 |
| EORTC QLQ-C30 Appetite loss  (0-100, higher values indicate higher burden) | 5.26 (5.51) | 1.66 (3.91) | -3.60 | 7.15 | -17.60 | 10.41 | 0.614853967 | 1 |
| EORTC QLQ-C30 Constipation  (0-100, higher values indicate higher burden) | 0.69 (5.05) | 1.11 (3.59) | 0.42 | 6.56 | -12.44 | 13.28 | 0.948908365 | 1 |
| EORTC QLQ-C30 Diarrhea  (0-100, higher values indicate higher burden) | 6.20 (5.20) | 4.94 (3.64) | -1.26 | 6.67 | -14.34 | 11.82 | 0.850158227 | 1 |
| SSD-12 Total score  (0-42, higher values indicate higher severity; subscales 0-16) | -0.54 (1.52) | -2.17 (1.07) | -1.63 | 1.96 | -5.48 | 2.22 | 0.406082274 | 1 |
| SSD-12 Subscale Cognitive aspects | 0.07 (0.59) | -0.04 (0.42) | -0.11 | 0.77 | -1.62 | 1.40 | 0.887680371 | 1 |
| SSD-12 Subscale Affective aspects | -0.37 (0.75) | -1.17 (0.53) | -0.80 | 0.97 | -2.70 | 1.11 | 0.412388833 | 1 |
| SSD-12 Subscale Behavioral aspects | 0.27 (0.59) | -0.96 (0.41) | -1.24 | 0.75 | -2.71 | 0.24 | 0.100603483 | 1 |
| NCCN Distress Thermometer  (0-10, higher values indicate higher severity) | -0.22 (0.52) | -1.25 (0.37) | -1.03 | 0.68 | -2.36 | 0.30 | 0.129740539 | 1 |

*T0 = baseline, T1 = after the waiting period, T2 = end of participation; SE: Standard Error; CI: Confidence Interval; LB: lower-bound; UB: upper-bound; q-value: adjusted p-value (Bonferroni-Holm); BIS: Body Image Scale; BMQ: Body Mindfulness Questionnaire; HADS: Hospital Anxiety and Depression Scale; SF-36: 36 Item Short Form Health Survey; EORTC: European Organization for Research and Treatment of Cancer; QLQ: Quality of Life Questionnaire; SSD-12: Somatic Symptom Disorder-B Criteria Scale; NCCN: National Comprehensive Cancer Network.*

**Supplementary Table 3.** Results from adjusted mixed-effect linear regression models contrasting T1 to T2 with T0 to T1 in the completer sample

| **Adjusted analyses**  (Completer, adjusted by gender and age) | Contrast (SE) | | Estimate | SE | 95% CI | | *p*-value | *q*-value |
| --- | --- | --- | --- | --- | --- | --- | --- | --- |
|  | T1 vs T0 | T2 vs T1 |  |  | LB | UB |  |  |
| Body Image Scale (BIS)  (0-30, higher values indicate higher burden) | -1.25 (1.24) | -0.37 (0.87) | 0.87 | 1.60 | -2.25 | 4.00 | 0.583913 | 1 |
| BMQ Experiencing body awareness  (7-42, higher values represent a better outcome) | -2.67 (1.43) | 0.51 (1.01) | 3.17 | 1.85 | -0.46 | 6.80 | 0.086878 | 1 |
| BMQ Appreciating body awareness  (7-42, higher values represent a better outcome) | -2.94 (1.31) | 4.90 (0.92) | 7.84 | 1.69 | 4.52 | 11.15 | 3.54E-06 | 0.0001 |
| HADS Anxiety  (0-21, higher values indicate higher severity) | -0.63 (0.65) | -0.65 (0.46) | -0.02 | 0.84 | -1.66 | 1.63 | 0.983162 | 1 |
| HADS Depression  (0-21, higher values indicate higher severity) | 0.25 (0.51) | -0.76 (0.36) | -1.02 | 0.65 | -2.29 | 0.26 | 0.118189 | 1 |
| SF-36 Vitality  (0-100, higher values represent a better outcome) | 2.35 (3.77) | 5.10 (2.64) | 2.76 | 4.84 | -6.73 | 12.25 | 0.569024 | 1 |
| SF-36 Mental Health  (0-100, higher values represent a better outcome) | -0.80 (3.47) | 2.50 (2.44) | 3.30 | 4.47 | -5.45 | 12.06 | 0.459597 | 1 |
| EORTC QLQ-C30 Global health status  (0-100, higher values represent a better outcome) | -3.07 (3.75) | 7.10 (2.67) | 10.17 | 4.86 | 0.64 | 19.70 | 0.036439 | 0.8881 |
| EORTC QLQ-C30 Physical functioning  (0-100, higher values represent a better outcome) | -0.61 (2.36) | 5.78 (1.66) | 6.39 | 3.04 | 0.43 | 12.35 | 0.035525 | 0.8881 |
| EORTC QLQ-C30 Role functioning  (0-100, higher values represent a better outcome) | 0.51 (5.86) | 6.30 (4.15) | 5.80 | 7.57 | -9.04 | 20.63 | 0.443915 | 1 |
| EORTC QLQ-C30 Emotional functioning  (0-100, higher values represent a better outcome) | -6.85 (5.19) | 2.29 (3.65) | 9.14 | 6.69 | -3.96 | 22.25 | 0.171463 | 1 |
| EORTC QLQ-C30 Cognitive functioning  (0-100, higher values represent a better outcome) | -6.26 (5.61) | 3.07 (3.94) | 9.33 | 7.22 | -4.82 | 23.48 | 0.196259 | 1 |
| EORTC QLQ-C30 Social functioning  (0-100, higher values represent a better outcome) | -7.78 (5.76) | 5.08 (4.05) | 12.86 | 7.41 | -1.67 | 27.39 | 0.082734 | 1 |
| EORTC QLQ-C30 Fatigue  (0-100, higher values indicate higher burden) | -3.77 (5.34) | -2.62 (3.76) | 1.16 | 6.87 | -12.32 | 14.63 | 0.86649641 | 1 |
| EORTC QLQ-C30 Nausea and vomiting  (0-100, higher values indicate higher burden) | 0.10 (4.16) | -1.49 (3.04) | -1.59 | 5.52 | -12.41 | 9.23 | 0.77336882 | 1 |
| EORTC QLQ-C30 Pain  (0-100, higher values indicate higher burden) | 2.89 (6.58) | 1.76 (4.66) | -1.13 | 8.51 | -17.82 | 15.55 | 0.894225923 | 1 |
| EORTC QLQ-C30 Dyspnea  (0-100, higher values indicate higher burden) | 0.70 (6.09) | -3.73 (4.29) | -4.44 | 7.80 | -19.73 | 10.86 | 0.569735102 | 1 |
| EORTC QLQ-C30 Insomnia  (0-100, higher values indicate higher burden) | 6.01 (7.92) | -4.19 (5.60) | -10.20 | 10.25 | -30.28 | 9.89 | 0.319692216 | 1 |
| EORTC QLQ-C30 Appetite loss  (0-100, higher values indicate higher burden) | 5.06 (5.53) | 1.61 (3.91) | -3.45 | 7.16 | -17.48 | 10.57 | 0.629463349 | 1 |
| EORTC QLQ-C30 Constipation  (0-100, higher values indicate higher burden) | 0.60 (5.06) | 1.09 (3.59) | 0.48 | 6.56 | -12.38 | 13.35 | 0.941172762 | 1 |
| EORTC QLQ-C30 Diarrhea  (0-100, higher values indicate higher burden) | 6.87 (5.20) | 5.12 (3.64) | -1.75 | 6.67 | -14.82 | 11.33 | 0.793161427 | 1 |
| SSD-12 Total score  (0-42, higher values indicate higher severity; subscales 0-16) | -0.41 (1.52) | -2.14 (1.07) | -1.73 | 1.96 | -5.58 | 2.12 | 0.377783249 | 1 |
| SSD-12 Subscale Cognitive aspects | 0.16 (0.59) | -0.03 (0.42) | -0.18 | 0.77 | -1.69 | 1.33 | 0.812635008 | 1 |
| SSD-12 Subscale Affective aspects | -0.32 (0.75) | -1.16 (0.53) | -0.84 | 0.97 | -2.74 | 1.06 | 0.388200645 | 1 |
| SSD-12 Subscale Behavioral aspects | 0.32 (0.58) | -0.95 (0.41) | -1.27 | 0.75 | -2.75 | 0.21 | 0.091738418 | 1 |
| NCCN Distress Thermometer  (0-10, higher values indicate higher severity) | -0.16 (0.52) | -1.23 (0.37) | -1.07 | 0.68 | -2.40 | 0.26 | 0.113954655 | 1 |

*T0 = baseline, T1 = after the waiting period, T2 = end of participation; SE: Standard Error; CI: Confidence Interval; LB: lower-bound; UB: upper-bound; q-value: adjusted p-value (Bonferroni-Holm); BIS: Body Image Scale; BMQ: Body Mindfulness Questionnaire; HADS: Hospital Anxiety and Depression Scale; SF-36: 36 Item Short Form Health Survey; EORTC: European Organization for Research and Treatment of Cancer; QLQ: Quality of Life Questionnaire; SSD-12: Somatic Symptom Disorder-B Criteria Scale; NCCN: National Comprehensive Cancer Network.*

**Supplementary Table 4.** Content of interventions: Group body psychotherapy with cancer patients and smartphone based bodily interventions

*Published in Grossert et al. (2019) that was distributed under the terms of the Creative Commons Attribution 4.0 International License (http://creativecommons.org/licenses/by/4.0/).*

|  | **Content group body psychotherapy** | | | | **Content audiofiles** |
| --- | --- | --- | --- | --- | --- |
| **Session** | **Topic** | **Opening**  **including short feedback on the previous session** | **Introduction & Exercise including sharing**   1. **Introduction and Psychoeducation** 2. **Strategies/Exercise** 3. **Sharing of own experiences** | **Closure**  **including perspective for the upcoming week** | **Smartphone based bodily interventions**  **supporting transfer from group BPT sessions into daily live** |
| **Duration** | **In Total 90 min** | **15-20 min** | **50-65 mins** | **10-15 min** | randomly on 3 days a week  10-15 min |
| 1. | **Group cohesion and body perception and awareness** | - Self-introduction - Expectations and fears | 1. - Reflection about bodily perception, body image, body disturbances and body work experience - Introduction of BPT terms 2. - Breath perception  - Body awareness with BodyScan technique, supine position - Self - contact (hands on/off) 3. Reflection about own experience during the exercises | What can I take with me after this first contact in the group? | Body awareness with body scan technique |
| 2. | **Bodily resources and**  **grounding/ anchoring** | - Short body scan CEB - Breath perception - Feedback | 1. - Body as a resource   - Balance between distress and resources, bodily stress reaction, adapted (Levine, 2011)   1. - Body awareness with BodyScan technique, standing position   - Foot work and anchoring with the focus on connection to stability, e.g. p97 (Kaul and Fischer, 2016) - Movement perception including mirroring   1. Reflection about own experience during the exercises | How to transfer exercise skills into daily life? | Anchoring/grounding with footwork using a small rubber ball |
| 3. | **Boundary awareness** | - Short body scan CEB - Anchoring exercise - Feedback | 1. Space and boundaries including importance of having the choice between own and shared space 2. Boundary awareness:   - Lika Breathing technique, p37 (Gauch Mühle et al., 2006)  - Boundary awareness: exploring own space and own boundaries, p84 (Kaul and Fischer, 2016)   1. Reflection about own experience during the exercises | Transfer of boundary awareness into daily life experience. | Boundary awareness through Lika breathing technique |
| 4. | **Impulses and social/group interaction** | - Short body scan CEB - Lika breathing technique, p37 (4) - Feedback | 1. Social interactions and (body) impulses to get into/out of social interactions 2. – Body self-release techniques, adapted p209 (Kaul and Fischer, 2016)   - Awareness of bodily perception and the nature of impulses  - nonverbal contact Interaction with different body parts   1. Reflection about own experience during the exercises | How can I find a witness position being aware of perception and impulses?  What do I need? | Relaxation through body self-release techniques |
| 5. | **Embodied emotions** | - Short body scan CEB - Anchoring exercise - Feedback | 1. Integration model of human experience, p26 (Kaul and Fischer, 2016) 2. - Mapping of feelings under the cancer disease and treatment   - Focus on embodiment: body sculptures of emotions, adapted (Tschacher et al., 2017)   1. Reflection about own experience during the exercises | How to become aware of feelings and how to explore and share them in daily life? | Self-awareness through ‘four body zone’ exercise |
| 6. | **Summary** | - Short body scan CEB - Free choice of exercise repetition - Feedback | 1. Summary and open questions 2. Free choice of exercise repetition 3. Closing: Ritual “*being connected while continue on individual path*” | What would I take with me?  What would I leave in this group/room?  Evaluation |  |

*BPT: Body Psychotherapy, CEB: cognition, emotions, body perception*

**References**

Gauch Mühle, R., Gross-Gstöhl, E., and Radelfinger, S. (2006). *Die Psychodynamik des Atems und des Meridiansystems zur Gesundheitsförderung und Therapie*. Uelzen: Medizinisch Literarische Verlagsgesellschaft.

Grossert, A., Meffert, C., Hess, V., Rochlitz, C., Pless, M., Hunziker, S., et al. (2019). A clinical trial of group-based body psychotherapy to improve bodily disturbances in post-treatment cancer patients in combination with randomized controlled smartphone-triggered bodily interventions (KPTK): study protocol. *BMC Psychol.* 7, 90. doi:10.1186/s40359-019-0357-1.

Kaul, E., and Fischer, M. (2016). *Einführung in die integrative Körperpsychotherapie IBP (Integrative Body Psychotherapy)*. Bern: Hogrefe Verlag.

Levine, P. A. (2011). *Sprache ohne Worte: Wie unser Körper Trauma verarbeitet und uns in die innere Balance zurückführt*. München: Kösel-Verlag.

Tschacher, W., Storch, M., Hüther, G., and Cantieni, B. (2017). *„Embodiment: Die Wechselwirkung von Körper und Psyche verstehen und nutzen*,“ *in Embodiment: Die Wechselwirkung von Körper und Psyche verstehen und nutzen, 3rd ed. W. Tschacher, M. Storch, G. Hüther, and B. Cantieni* (Bern: Hogrefe, vorm. Verlag Hans Huber), p 184.
